# How Are We Prescribing? a Re-Audit of Prescribing Practices for People With Personality Disorder Presented at the Local Personality Disorder Forums

**DOI:** 10.1192/bjo.2022.453

**Published:** 2022-06-20

**Authors:** Maham Khan, Neelima Reddi

**Affiliations:** Surrey and Borders Partnership NHS Trust, Leatherhead, United Kingdom

## Abstract

**Aims:**

NICE recommends that drug treatments shouldn't be used specifically for the treatment of individual symptoms or behaviours associated with Borderline Personality Disorder but may be considered for the overall treatment of comorbid conditions. National audits have been completed by Prescribing Observatory for Mental Health UK (POMH-UK) in 2012 and 2014, with a local Trust audit in 2017. Subsequently, ‘Guidance for Prescribing in Personality Disorder’ was published and circulated across the Trust in Dec 2020.This audit aims to establish whether there has been a change in the prescribing trends in the Trust since the last audit in 2017.

**Methods:**

Electronic records of patients identified and presented at Personality Disorder forums throughout the Trust between June-December 2021 were reviewed.

Two audit standards and four treatment targets derived from the POMH-UK audit were used.

**Results:**

24 electronic patient records were reviewed.

79% patients had a documented crisis plan, fewer than the 87% in 2017.

The proportion on antipsychotics with documented clinical reasons for prescribing was 69%, compared to 43% in 2017.

For those on antipsychotics in the absence of a comorbid psychotic illness, 91% were on them for >4 weeks, compared to 86% in 2017.

Z-hypnotics were prescribed for >4weeks in 37.5%, significantly more than the 13% in 2017. Benzodiazepines were prescribed for >4 weeks in 38% of patients, with 28% recorded in 2017.

100% of patients eligible for medication reviews had had them, an improvement from 90%in 2017.

**Conclusion:**

Compared to the previous audit, fewer patients had crisis plan documentation, but more patients had a clinical indication for antipsychotics recorded.

Rates of prescribing Z-hypnotics, benzodiazepines and antipsychotics for >4 weeks seems to have risen, demonstrating a lower compliance with the treatment targets.

The proportion of patients having medication reviews has improved, however, the quality of these reviews remains similar to the 2017 audit.

The findings will be presented within the Trust with the re-emphasis on guidelines.

Prescribing in people with personality disorders can be revisited in 2 years.

